# *Hii Chii Kok v (1) Ooi Peng Jin London Lucien; (2) National Cancer Centre*: Modifying Montgomery

**DOI:** 10.1093/medlaw/fwy044

**Published:** 2019-01-28

**Authors:** Louise V Austin

**Affiliations:** Centre for Health, Law, and Society, University of Bristol School of Law, Bristol, UK

**Keywords:** Informed consent, standard of disclosure, Montgomery standard

## Abstract

In *Hii Chii Kok v (1) Ooi Peng Jin London Lucien; (2) National Cancer Centre*, the Singapore Court of Appeal followed the approach of other Commonwealth jurisdictions by rejecting the application of *Bolam* as the standard of disclosure in claims concerning informed consent to medical treatment. Instead, the court employed a modified version of the standard of disclosure adopted in *Montgomery v Lanarkshire Health Board*. While broadly welcomed, *Montgomery* has been criticised for its lack of clarity on the application of some elements of its disclosure standard. In particular, questions remain as to: what factors should be taken into account within the reasonable and particular patient limbs of the test of materiality; how will the ‘reasonableness’ of alternative treatments be determined; and what is the scope of the therapeutic exception. This case commentary explores how *Hii’s* analysis of the modified standard offers insights into how those elements of *Montgomery* could be interpreted in the future.

## INTRODUCTION

In 2017, the Singapore Court of Appeal followed the approach of other Commonwealth jurisdictions by rejecting the application of *Bolam*^1^ to claims concerning informed consent. In *Hii Chii Kok v (1) Ooi Peng Jin London Lucien; (2) National Cancer Centre* (‘*Hii’*),^2^ the Singapore court said that the *Bolam* standard was doctor-centred as it focused upon whether a responsible body of medical opinion would have given the advice in question.^3^ The need to obtain a patient’s informed consent to medical treatment, however, arose from the central principle that the patient had autonomy to decide such matters. In recognition of this, the Court concluded it was necessary to move towards a more patient-centric standard of disclosure.^4^ A similar conclusion had previously been reached in the UK in the landmark case of *Montgomery v Lanarkshire Health Board* (‘*Montgomery*’), which determined that the adequacy of disclosure was to be judged from the perspective of the patient, rather than from the perspective of clinicians.^5^

Whilst the court in *Hii* recognised the need to move towards a patient-centric standard of disclosure, they saw the medical profession as still having a role to play in assessing what information should be given to patients.^6^ The Singapore Court of Appeal thus adopted a modified version of the *Montgomery* test, utilising a three-stage approach, that is discussed below. While the decision in *Montgomery* was largely welcomed by academic scholars,^7^ the judgment has been criticised for its lack of clarity on some elements of the standard. Although the form of the standard of disclosure in *Hii* is distinct from the *Montgomery* standard, its substance is broadly the same. As such, the analysis of the different stages in *Hii* can be used to shed light upon how aspects of the *Montgomery* standard could be interpreted. The Singapore decision is not binding on UK courts, but given that the development of the *Montgomery* standard was influenced by judgments in Australia, Canada, and the USA, *Hii* could be used to influence future interpretations of *Montgomery*.

I begin with a summary of the *Hii* case and the different standards of disclosure set out in *Montgomery* and *Hii*. Utilising the analysis of the Singapore Court of Appeal, I then explore the different stages of the *Hii* standard, highlighting how this analysis can be used to interpret elements of the *Montgomery* standard. In particular, I focus upon: the distinction between a reasonable person in the patient’s circumstances and the particular patient; defining reasonable alternative treatments; the role *Bolam* may continue to play; the scope of the therapeutic privilege exception; and how information should be communicated. My analysis illustrates that whilst *Hii* aids the interpretation of some of these issues, the role *Bolam* may play in future cases remains unclear, despite the Supreme Court’s unequivocal rejection of it in *Montgomery*. There also remains a lack of guidance for doctors on *how* information should be communicated.

## HII: MODIFYING MONTGOMERY

### Background

In 2010, following the identification of a neuroendocrine tumour (NETs) in his right lung, Mr Hii underwent a Gallium scan^8^ for the purposes of assessing other nodules present in his lung. The Gallium scan, however, also identified the existence of two nodules in his pancreas; one in the body of the pancreas and one in the pancreas head. His treating clinicians believed those nodules may be pancreatic NETs (PNETs) and an MRI scan was performed to investigate this further. The MRI scan revealed no pancreatic masses, suggesting the pancreatic lesions seen on the earlier scan were not malignant. However, in light of the conflicting findings, his clinicians could not be certain as to the true nature of the pancreatic nodules.

Mr Hii was advised of the scan results and the uncertainty of the diagnosis. However, in light of the possibility that the pancreatic nodules could be cancerous, the first defendant, Dr Ooi, advised Mr Hii to undergo surgery to remove the nodules. Unfortunately, the position of one of the nodules in the pancreas head meant that Mr Hii would have to undergo a Whipple procedure. This procedure involved removing the head of the pancreas, together with parts of the bile duct, gallbladder, duodenum, and stomach. Following this, the remaining parts of the pancreas, bile duct and stomach would be surgically joined to preserve the integrity of the gastrointestinal tract (an ‘anastomosis’). Mr Hii was advised that the surgery was complex and carried a risk of post-operative anastomotic breakdown.

Given the seriousness and complexity of the proposed surgery, Mr Hii explored the possibility of further investigations to determine the nature of the pancreatic nodules. Those discussions took place separately with each defendant. During those discussions, Mr Hii was advised of four investigation/treatment options:

1. wait 6 months and then undergo further scanning. There was a risk that if the lesions were cancerous, the malignancy would spread during this time.
2. Undergo further investigations by way of ultrasound examination and biopsy of the masses. There was a risk these investigations would be inconclusive as a negative finding would not confirm the lesions were non-cancerous.
3. Undergo surgical removal of the lesion in the body of the pancreas and biopsy the lesion in the head of the pancreas. This would avoid the need for the Whipple procedure but, subject to the findings of the biopsy, further surgery at the head of the pancreas may have been required at a later date.
4. Undergo surgical removal of both lesions by way of the Whipple procedure.

Dr Ooi advised Mr Hii to undergo the Whipple procedure in light of the uncertainty of diagnosis; the risk that further investigations would not resolve that uncertainty; and that delay could result in cancerous spread. Dr Ooi also reassured Mr Hii that, although there was a risk of anastomotic breakdown, given Mr Hii’s age and Dr Ooi’s experience he was a good candidate for the procedure. Mr Hii eventually accepted this advice and underwent the surgery in August 2010.

Post-operatively, it was confirmed the pancreatic nodules were not cancerous. Unfortunately, in the post-operative period, Mr Hii’s anastomosis broke down leading to the need for more surgery during which further parts of his pancreas were removed, together with his spleen.

Mr Hii subsequently brought claims against Dr Ooi and the NCC alleging negligence in their diagnosis, advice and treatment. His claims were dismissed by the Singapore High Court and that decision was upheld on appeal.^9^ This commentary focuses on the aspects of the Court of Appeal’s judgment dealing with the alleged negligent advice.

In summary, Mr Hii argued that:

1. *Montgomery* reflected the correct standard of care for claims of negligent medical advice.
2. Applying *Montgomery*, the advice of both defendants was deficient because:
  - they failed to advise him of the limitations of the Gallium scan and the importance of the absence of positive results on other scans;
  - they should have advised him to undergo an ultrasound examination and biopsy before proceeding to surgery by way of the Whipple procedure;
  - he was misinformed when he was told negative results from other scans could not rule out PNETs and were, therefore, not of diagnostic value;
  - they should have advised him to undergo surgery to remove the pancreas body nodule while conducting further tests on the nodule in the pancreas head.
3. Had he been properly advised, he would not have undergone the Whipple procedure.^10^

Both the High Court and the Court of Appeal concluded he had been given proper advice on the uncertainty of the diagnosis and the available diagnostic/treatment options. Having made that finding, the Singapore Court of Appeal declined to consider the issue of causation.^11^ In giving its ruling, the Court of Appeal did take the opportunity to clarify the legal standard of disclosure in cases of alleged negligent medical advice.

### Applicable Law pre-Hii

Prior to the decision in *Hii*, in *Gunapathy* the Singapore Court of Appeal had accepted that the *Bolam* test applied to disclosure of risks in the medical context.^12^ That test derived from the English case of *Bolam v Friern Hospital Management Committee* where MacNair J held that a doctor would not be negligent if he or she had acted *‘in accordance with a practice accepted as proper by a responsible body of medical men skilled in that particular art’*.^13^ The later English case of *Bolitho* held that a body of opinion would not be responsible if it was ‘*not capable of withstanding logical analysis’.*^14^ In *Montgomery*, however, the UK Supreme Court had confirmed that *Bolam* was no longer the applicable standard of disclosure in medical treatment.^15^ Mr Hii argued that *Montgomery* also reflected the correct approach in Singapore.

### The Montgomery Standard

In *Montgomery*, having rejected *Bolam* as the applicable standard for determining the question of negligent disclosure in medical treatment, the UK Supreme Court set out the correct standard of disclosure. The court held that doctors had a duty to disclose the material risks and anticipated benefits of recommended treatment, together with any reasonable alternative or variant treatments, and the risks and benefits of such alternatives.^16^ A risk would be *‘material’* if:

*‘in the circumstances of the particular case, a reasonable person in the patient’s position would be likely to attach significance to the risk, or the doctor is or should reasonably be aware that the particular patient would be likely to attach significance to it.’* ^17^

However, doctors would be justified in withholding information about material risks from a patient if: (a) the doctor reasonably considered that disclosure would be seriously detrimental to the patient’s health (‘the therapeutic exception’);^18^ (b) the circumstances made non-disclosure necessary, for example, where treatment was required urgently but the patient was unconscious or otherwise unable to make a decision;^19^ or (c) the patient did not wish to be informed of risks.^20^

### Montgomery Modified

The Singapore High Court held that they were bound by *Gunapathy*, but further found that Mr Hii’s claim based on negligent disclosure and advice failed whether *Bolam* or *Montgomery* was applied.^21^ Although that decision was upheld by the Court of Appeal, the court took the opportunity to clarify the position as to what the standard of care was in the context of medical advice.

The court held that diagnosis, advice and treatment were different aspects of a doctor’s duty of care to the patient, and thus the application of different tests to determine the standard of care for each aspect was justified. *Bolam* should continue to apply to diagnosis and treatment but this was a doctor-centred test. Given the recognition that patients have autonomy to decide whether to undergo medical treatment or not, the question of whether a patient had received sufficient information to enable them to participate meaningfully in that decision had to be viewed from the patient’s perspective. However, doctors’ views remained relevant to the question of what treatments were available, the risks and benefits of those, and the impact they may have upon the patient. The standard of disclosure should seek to strike an appropriate balance between the perspectives of the patient and the doctor and to this end, the Court of Appeal adopted a modified version of the *Montgomery* standard aimed at striking that balance.^22^

The modified approach involved a three-stage process in assessing whether the standard of disclosure had been met, with each stage viewed from the perspective of the court as the ultimate arbiter of such disputes.

1. The patient had to:
  - Identify the exact nature of the information it was alleged had not been provided;
  - Establish why that information was relevant and material.
2. If (1) was met, the court had to determine whether the doctor was in possession of the information in question.
3. If the doctor did possess the information, the court would then examine the doctor’s reasons for withholding that information in order to determine whether the non-disclosure was justified.^23^

## BREAKING DOWN THE *HII* STANDARD

The *Hii* standard is framed differently to the *Montgomery* standard as the *Hii* standard focuses on the approach the court must take when determining whether non-disclosure in the context of medical treatment amounts to negligence. In contrast, the *Montgomery* standard is framed from the perspective of what the doctor must do. Despite these differences in form, the two standards are similar in substance. The Court of Appeal’s judgment in *Hii* analysed its three-stage approach with a view to giving practical guidance on its application. This analysis highlights the similarities between the two standards and sheds light on how elements of the *Montgomery* standard could be interpreted.

### Stage One: Relevant and Material Information

Stage one of the *Hii* standard requires disclosure of:

*‘(a) information that would be relevant and material to a reasonable patient situated in the particular patient’s position, or (b) information that a doctor know is important to the particular patient in question.’* ^24^

This echoes the *Montgomery* test for determining which risks should be disclosed. In *Montgomery* it was said doctors must disclose material risks and a risk would be material if: (a) a reasonable person in the patient’s circumstances would have attached significance to it; or (b) where the doctor knew, or ought reasonably to have known, that the particular patient would attach significance to it.

Heywood and Miola have highlighted *Montgomery’s* failure to clarify what factors fall to be considered under the reasonable patient limb given the references to someone *‘in the patient’s position’*, and what factors would fall within the particular patient limb.^25^ Miola had previously highlighted this uncertainty in the context of the Australian case of *Rogers v Whittaker*,^26^ which formed the basis of the revised standard of disclosure in *Montgomery*. Miola noted that *Rogers* appeared to interpret *‘the patient’s position’* narrowly, limiting it to the patient’s physical characteristics.^27^ Thus, Heywood and Miola suggested that the patient’s physical characteristics would fall within the reasonable patient limb, whereas the patient’s *personal* characteristics (such as the patient’s views, eccentricities, and religious beliefs) would fall within the particular patient limb.^28^ The court in *Hii* appeared to have this criticism in mind^29^ as its judgment sought to clarify what information about the patient should be taken into account under each limb. However, the court adopted a different position to Heywood and Miola.

According to the *Hii* judgment, the patient’s personal circumstances would be relevant under the ‘reasonable patient’ limb. Thus, if a procedure carried a slight risk of scarring and the patient was an aspiring model, that risk should be disclosed on the basis that a reasonable patient who aspired to be a model would attach significance to that risk.^30^ In contrast, the particular patient limb would only trigger a duty of disclosure where the patient had asked particular questions, or expressed particular concerns.^31^

While the *Hii* judgment provides the desired clarity, the likelihood of this interpretation of the two limbs being extended to the *Montgomery* standard is uncertain. In particular, the Supreme Court in *Montgomery* rejected the notion that a patient had to ask specific questions to trigger disclosure beyond that which all patients would be entitled to. The court said that to hold otherwise would be:

*‘[…] a reversal of logic: the more a patient knows about the risks she faces, the easier it is for her to ask specific questions about those risks […] but it is those who lack such knowledge […] who are in the greatest need of information.’* ^32^

In addition, they said that such a requirement ignored *‘the social and psychological realities’*^33^ of the doctor–patient relationship where *‘few patients do not feel intimidated or inhibited to some degree’*.^34^

At first sight, this seems to suggest that the particular patient limb of *Montgomery* could not be interpreted as it was in *Hii* because to do so would require patients to ask questions to trigger disclosure. However, I argue that the *Hii* interpretation enables the test of materiality to address the disclosure needs of both the questioning and the unquestioning patient, while clarifying where particular factors about a patient fall to be considered. This is because, under the *Hii* interpretation, the need to disclose information to a patient who asks no questions and expresses no concerns is still triggered by reference to the patient’s perspective, rather than the doctor’s perspective. It takes account of the patient’s particular circumstances, which incorporates matters beyond the patient’s physical characteristics, such as the nature of the patient’s employment, religious beliefs etc. For patients, however, who do ask questions or express concerns, the particular patient limb then ensures that a duty of disclosure is triggered in response to those questions or concerns. Support for this interpretation can be found within Heywood and Miola’s paper where, in the context of the particular patient limb, they comment that *‘it is difficult to see how else a doctor might be found liable for something they were not told.’^35^*

### Stage One: Reasonable Alternative or Variant Treatments

Like *Montgomery*, *Hii* recognises that patients need to be advised of any reasonable alternative or variant treatments that are available, and their associated and comparative risks and benefits in order to weigh up how to proceed.^36^ Heywood and Miola have criticised *Montgomery* for failing to clarify how ‘reasonableness’ is to be assessed in this context. They note that two cases prior to *Montgomery* did consider the question of alternative procedures and treatments.^37^ Based on those earlier cases, Heywood and Miola said we could deduce the following principles: (1) there is an obligation to discuss alternative procedures where there is a less risky alternative that would be recommended by another healthcare professional; (2) there is no obligation to discuss alternative diagnoses. However, they said that the failure to consider those cases in *Montgomery* meant that there was a lack of guidance as to when an alternative or variant treatment would require disclosure.^38^

Laing suggested the uncertainty around what amounts to a reasonable alternative treatment was likely to be particularly problematic for practitioners issuing pharmacological prescriptions. In those circumstances, there may be a wide range of alternative medications available, all of which would have different risks, benefits and interactions. However, information about these may not be easily accessible.^39^

The court in *Hii* did give some guidance as to the potential scope of ‘reasonable alternative treatment’. The judgment stated that clinicians are not obliged to discuss ‘fringe’ treatments, alternative medicine, or *‘mainstream treatment options which are obviously inappropriate on the facts’*.^40^ However, this phrasing seems to point towards the application of a *Bolam* standard. How will the courts know whether a treatment was fringe, alternative, or mainstream but inappropriate without recourse to expert medical evidence? While the court in *Hii* did not seek to exclude the doctor’s perspective from information disclosure, they were clear (as was the court in *Montgomery*) that *Bolam* should not play a role.^41^

Thus, while *Hii* could fill the gap in guidance left by *Montgomery* as to what constitutes a ‘reasonable’ alternative, it seems it can only do so by reintroducing *Bolam*. Thus, despite *Montgomery’s* rejection of *Bolam* as the applicable standard for information disclosure in medical treatment, qualifying the standard by reference to ‘reasonableness’ makes it inevitable that *Bolam* will continue to play a role. Medical expert evidence will be necessary to determine what other treatments existed and the extent to which they were reasonable alternatives. Where there are differences of opinion on these between healthcare professionals, given that these involve questions of appropriate medical treatment, it is likely the courts will fall back on the *Bolam* approach. If this is to be done, it is important for the courts to be clear and explicit as to when reliance on *Bolam* will be justified and the boundaries of its use in order to ensure *Bolam* does not creep back in as the standard for information disclosure.^42^

### Stage Two: Possessing Information

If the patient is able to establish that the information in question was relevant and material, the court should then consider whether that information was in the doctor’s possession. This step is not explicitly addressed within the *Montgomery* standard but it is implicit in it. As was said in *Hii*, if the doctor did not have the information then it made ‘*little sense to ask whether* [the doctor] *should have given it to the patient; one cannot give what one does not have’*.^43^

Not being in possession of the information does not mean the doctor escapes liability. Instead, in *Hii* it was said that the court would have to consider whether the doctor *ought* to have been in possession of the information but that question was to be assessed under the rubric of negligent diagnosis or treatment where the appropriateness of the doctor’s conduct would be judged according to the *Bolam* standard.^44^ This was because, according to the court in *Hii*, *‘the crux of such a complaint will be that the doctor made the wrong diagnosis or failed to administer the proper treatment due to his ignorance or carelessness’*.^45^ However, it is submitted that this reasoning is flawed.

If the court finds that a relevant and material risk was not disclosed to a patient but the doctor’s reason for non-disclosure was lack of knowledge of the risk, then according to *Hii* this should be treated as a matter of negligent diagnosis or treatment. The presence of the risk, however, does not affect the diagnosis. In those circumstances then, it seems the court is saying that the treatment is negligent because the doctor did not make himself aware of the risk. This, however, only impacts treatment if the patient would have elected another form of treatment, or no treatment, if warned of the risk. The first question to determine will be whether the doctor should have known of the risk; and that will be judged by the *Bolam* standard. Assuming the doctor should have known of the risk, the court will then have to explore whether the patient would have proceeded with the treatment if the risk had been disclosed. However, that is a question of what effect the doctor’s failure to apprise himself of the risk had on the patient’s decision to proceed; it does not render the treatment, nor the doctor’s recommendation of that treatment, negligent. It is still a question of negligent advice and it seems the court’s desire to recategorise it as negligent diagnosis or treatment stems from a desire to keep the *Bolam* standard away from questions of disclosure. As with reasonable alternative treatments however, it highlights the risk of the reintroduction of *Bolam* to determine questions of reasonableness.

### Stage Three: The Therapeutic Privilege Exception

*Hii* recognised that there would be some circumstances where non-disclosure of material information would be justified. Those circumstances were not limited and would depend upon the court’s assessment of the doctor’s reasons for non-disclosure. Examples included: the patient choosing not to be informed; emergencies where treatment was necessary to avoid the patient’s death or serious harm, yet the patient lacked temporary decision-making capacity, which are practical issues; or, more controversially, the therapeutic privilege, whereby the doctor believed giving the patient particular information would cause the patient serious physical or mental harm.^46^*Montgomery* also recognised these as scenarios which could justify non-disclosure, although the Supreme Court referred to them as ‘exceptions’ to the requirement of disclosure, rather than as justifications for non-disclosure.^47^

In the wake of *Montgomery*, Heywood and Miola and Cave have noted the failure of the Supreme Court to clarify the scope of the therapeutic privilege.^48^ For example, what amounts to ‘serious harm’ or ‘detriment’ to the patient’s health is not defined.^49^ There is little guidance to be derived from case law as *‘few cases refer to the therapeutic privilege, even fewer apply it and none have accepted it as a defence’*.^50^

*Hii* does seek to define the scope of the therapeutic privilege with the judgment stating that the privilege covers those who *‘have mental capacity [but whose] decision-making abilities are impaired to an appreciable degree’*.^51^ This includes: those with anxiety disorders; geriatric patients who are *‘easily frightened out of having even relatively safe treatments that can drastically improve their quality of life’*;^52^ or those whose intellectual abilities or education make it extremely difficult to *‘explain the true reality’* to them.^53^ This seems to move towards a territory whereby capacity as a condition of autonomous decision-making falls by the wayside as the therapeutic privilege is used to justify non-disclosure in cases where people have capacity but may refuse treatment if given relevant and material information. However, as the Supreme Court did in *Montgomery*,^54^ the Court of Appeal in *Hii* said it is not to be used to enable doctors to prevent patients from making choices about medical treatment where they are capable of doing so, simply because the doctor considers treatment to be in their best interests.^55^ What is unclear is where the line is drawn in scenarios of ‘borderline’ capacity where a patient’s decision-making is impaired, yet not to the extent that the patient is deemed incapable of making a decision.

In *Hii* the court said that in determining whether the therapeutic privilege applies, medical expert evidence would be relevant although the *Bolam* standard would not be applied. The question for the court is not whether a body of doctors supported non-disclosure but whether:

*‘the patient was suffering from such an affliction that he in fact was likely to be harmed by being apprised of the relevant information. Or that the patient, though not strictly lacking mental capacity, nonetheless suffered from such an impairment of his decision-making abilities that the doctor would be entitled to withhold the information […].’* ^56^

Despite this clarification in *Hii*, given the absence of cases utilising the therapeutic privilege as a defence, the need for its existence remains open to question.^57^ It is notable that, despite *Montgomery’s* approval of the therapeutic privilege, in the General Medical Council’s proposed revised consent guidance, the therapeutic privilege is absent, suggesting that that this is no longer seen as a necessary exception in medical practice.^58^

### How to Communicate

At first sight, *Hii* appears to take a different stance to *Montgomery* in respect of the doctor’s role in securing patient understanding. While *Montgomery* said that the doctor’s advisory role involved dialogue aimed at *ensuring* that patients understood the information they were given,^59^ in *Hii* it was said that the doctor’s obligation was *not* to ensure understanding. Instead, it was limited to the doctor taking *reasonable steps* to enable the patient to understand the information.^60^ Thus, Montgomery seems to place doctors under a more onerous obligation in respect of ensuring patient understanding. However, given that a doctor’s duty in negligence is to take reasonable care, it seems likely that doctors will only need to take reasonable steps to ensure understanding, bringing the *Montgomery* standard closer to the *Hii* interpretation.^61^

Heywood and Miola note *Montgomery’s* direction to ensure understanding through dialogue, but are critical of its failure to set out what practical steps must be taken and how far doctors are expected to go to ensure understanding.^62^*Hii* does explore the nature of communication that should take place, stating that provision of information alone is not enough and it must be *‘accompanied by a quality of communication that is commensurate with the ability of the patient to understand the information’*.^63^ Thus, the information must be presented ‘*in terms and at a pace’*^64^ that allows the patient to assimilate it in order to make an informed decision. This suggests that just as the doctor has to take account of the individual patient’s circumstances when deciding *what* to disclose, the doctor must also take account of the patient’s needs when deciding *how* to inform the patient. There remains, however, a lack of clarity about how this should be done in an individual case. For example, would it be sufficient to discuss the risks of an operation with a patient and then provide an information leaflet setting out those risks for the patient to take away, with the patient being given the option to contact the doctor for further information if there is anything the patient doesn’t understand? Or should there be a second consultation as a matter of routine to check understanding? If so, how should those checks take place? These are questions that remain unanswered by either *Montgomery* or *Hii* yet will be important for doctors to know in clinical practice.

## CONCLUSION

*Hii* is the latest in a line of Commonwealth cases to reject the application of the *Bolam* standard to cases of informed consent to medical treatment in favour of a patient-centric standard. In doing so, it echoed the approaches of other Commonwealth jurisdictions (namely Australia, the USA and Canada) by recognising that while the adequacy of disclosure should be judged from the patient’s perspective, the medical profession’s views would still be relevant (although not determinative). This stands in contrast to *Montgomery* which stated there was no longer any role for *Bolam* in such cases and, by implication, the views of the medical profession. This led the Singapore court in *Hii* to adopt a modified version of the *Montgomery* test in which it sought to balance medical and patient perspectives in questions of informed consent. Despite this, there are substantive similarities between the *Hii* and *Montgomery* standards which enable us to draw upon the analysis in *Hii* in order to shed light on questions raised by the revised standard in *Montgomery*. *Hii* offers some approaches to interpretation which could be used to develop the application of the *Montgomery* standard in the UK, but gaps remain.

The analysis in *Hii* (and that in other Commonwealth jurisdictions) suggests that the views of the medical profession will continue to play a role in determining whether an alternative treatment amounted to a reasonable alternative and whether a doctor should have been in possession of particular information. Where there is disagreement within the medical profession about these questions, it seems inevitable that *Bolam* will creep back in as a way of resolving such disputes, even if it is not determinative of the ultimate question of the adequacy of disclosure. It is important that the UK courts recognise this when applying *Montgomery* and explicitly set out when and to what extent medical professional views have influenced decisions. The use of such views should be limited to questions which can only be resolved with medical opinion and should not be allowed to influence the court’s view as to what information the patient would have been likely to have found significant.

There is a persisting lack of clarity as to how the therapeutic privilege or exception should be applied and its role in cases of borderline capacity, given that it should not be used to override a patient’s choice which may run counter to the clinician’s view as to what is best for the patient. This lack of clarity and the absence of cases however suggest this may be a theoretical problem rather than a practical one. Given the GMC’s decision to remove reference to it in their consent guidance, it may be time for the UK courts to consider at the next opportunity whether such an exception is needed at all and, if so, what its scope should be.

Finally, there remain unanswered questions as to what practical steps doctors are expected to take when communicating information to patients to enable and ensure that patients understand that information. The qualification of ‘reasonableness’ sheds little light on this. It seems unlikely it will be enough to do as other doctors would do as *Hii* suggests the need to tailor communication to the individual; an approach that accords with *Montgomery’s* focus on the individual patient. Further clarification on this could be developed in conjunction with doctors, patients and medical professional bodies, such as the GMC.

*Hii* then offers some welcome insights into *Montgomery’s* unanswered questions but further clarity is needed in particular areas. Reviewing and monitoring approaches of the UK courts and those of other Commonwealth jurisdictions could provide that clarity.

## Footnotes

1
*Bolam v Friern Hospital Management Committee* [1957] 1 WLR 582.

2
*Hii Chii Kok v (1) Ooi Peng Jin London Lucien; (2) National Cancer Centre of Singapore Pte Ltd* [2017] SGCA 38.

3
*Bolam* (n 1) 587.

4
*Hii* (n 2) [4].

5
*Montgomery v Lanarkshire Health Board* [2015] UKSC 11.

6
*Hii* (n 2) [4].

7 Clark Hobson, ‘No (,) More Bolam Please: Montgomery v Lanarkshire Heath Board’ (2016) 79(3) MLR 488; Anne Maree Farrell and Margaret Brazier, ‘Not so New Directions in the Law of Consent? Examining *Montgomery v Lanarkshire Health Board*’ (2016) 42 JME 85; Rob Heywood and Jose Miola, ‘The Changing Face of Pre-Operative Medical Disclosure: Placing the Patient at the Heart of the Matter’ (2017) 133 LQR 296.

8 A Gallium scan uses radioactive material to look for inflammation, infection or cancer in the body: <https://medlineplus.gov/ency/article/003450.htm> accessed on 28 October 2018. In Mr Hii’s case it was used in combination with CT scanning: *Hii* (n 2) [8].

9
*Hii* (n 2) [1].

10 ibid [47].

11 ibid [201]–[203].

12
*Dr Khoo James and another v Gunapathy d/o Muniandy and another* [2002] SGCA 25.

13
*Bolam* (n 1) 587 per McNair J.

14
*Bolitho v City & Hackney Health Authority* [1998] AC 232, 243 per Lord Browne-Wilkinson.

15
*Montgomery* (n 5) [86].

16 ibid [87], [90].

17 ibid [87] per Lords Kerr and Reed. In formulating this test, the Supreme Court explicitly adopted the approach to materiality developed by the Australian High Court in *Rogers v Whitaker* (1992) 175 CLR 479 [16].

18
*Montgomery* (n 5) [88], [91].

19 ibid [88].

20 ibid [85].

21
*Hii* (n 2) [1].

22 ibid [4].

23 ibid [132]–[134].

24 ibid [132].

25 Heywood and Miola (n 7) 296, 304.

26
*Rogers* (n 17).

27 José Miola, ‘On the Materiality of Risk: Paper Tigers and Panaceas’ (2009) 17 Med Law Rev 76, 92.

28 Heywood and Miola (n 7) 304.

29 Miola (n 27) was cited in *Hii* (n 2).

30
*Hii* (n 2) [144]. The example the court gave was taken from: Rob Heywood, ‘Subjectivity in Risk Disclosure: Considering the Position of the Particular Patient’ (2009) 25(1) Professional Negligence 3, 7.

31
*Hii* (n 2) [145].

32
*Montgomery* (n 5) [58].

33 ibid.

34 ibid.

35 Heywood and Miola (n 7) 305.

36
*Hii* (n 2) [142].

37
*Birch v University College London NHS Foundation Trust* [2008] EWHC 2237 (QB); (2008) 104 BMLR 168: The patient suffered a stroke after a cerebral catheter angiogram was performed. She had not been advised that she could undergo an MRI scan instead which was less precise but carried no risk of stroke. Her claim was successful; *Meiklejohn v St George’s Healthcare NHS Trust* [2014] EWCA Civ 120; [2014] Med LR 122: The patient developed avascular necrosis, a recognised complication of medication he had been prescribed. He argued that he should have been advised of the alternative diagnoses and, therefore, alternative treatments. His claim was unsuccessful.

38 Heywood and Miola (n 7) 307–09.

39 Judy Laing, ‘Delivering Informed Consent Post-Montgomery: Implications for Medical Practice and Professionalism’ (2017) 33(2) PN 128, 143.

40
*Hii* (n 2) [142].

41 ibid [126]; *Montgomery* (n 5) [85]–[86], [115].

42 Other ‘non-*Bolam*’ informed consent cases have recognised that medical professional opinion will continue to be relevant but that it should not be determinative: *Canterbury v Spence* (1972) 464 F 2d 772 [27]; *Reibl v Hughes* [1980] 2 SCR 880, 895. This view was also expressed in an Australian case pre-dating *Rogers* (n 17): *F v R* (1983) 33 SASR 189, 194, 205.

43
*Hii* (n 2) [133].

44 ibid [133], [147].

45 ibid [133].

46 ibid [148]–[152].

47
*Montgomery* (n 5) [85], [88]. Such exceptions have also been recognised in other ‘non-*Bolam*’ informed consent cases: *Canterbury* (n 42) [37]; *Reibl* (n 42) 827; *Rogers* (n 17) [14], [16].

48 Heywood and Miola (n 7) 311; Emma Cave, ‘The Ill-informed: Consent to Medical Treatment and the Therapeutic Exception’ (2017) *CLWR* 46(2) 140, 142.

49 Heywood and Miola (n 7) 311.

50 Cave (n 48) 143.

51
*Hii* (n 2) [152].

52 ibid [152].

53 ibid [152].

54
*Montgomery* (n 5) [91].

55
*Hii* (n 2) [153].

56 ibid [153].

57 Cave (n 48) 155–156.

58 General Medical Council, ‘Decision making and consent. Supporting patient choices about health and care. Draft guidance for consultation’ (2018) <https://www.gmc-uk.org/-/media/ethical-guidance/related-pdf-items/consent-daft-guidance/consent-draft-guidance.pdf?la=en&hash=B117C71B5F6082312DD4C4A78EE6B39B9B43A8FB> accessed on 1 November 2018.

59
*Montgomery* (n 5) [90].

60
*Hii* (n 2) [154].

61 This would be consistent with ruling in *Al Hamwi v Johnston* [2005] EWHC 2006 (QB); [2005] All ER (D) 278 (Feb), yet *Al Hamwi* said doctors were not under a duty to ensure understanding which runs contrary to the dicta in *Montgomery* (n 5). *Al Hamwi* was not cited or considered in *Montgomery*.

62 Heywood and Miola (n 7) 311.

63
*Hii* (n 2) [155].

64 ibid [156].

